# Docetaxel enhances Vβ-directed T-cell activation and antitumor immunity mediated by a bifunctional TCR agonist in breast and prostate cancer models

**DOI:** 10.3389/fimmu.2026.1850760

**Published:** 2026-06-03

**Authors:** Jonelle K. Lee, Kellsye P. Fabian, Ginette S. Santiago-Sanchez, Francesca Rosato, Michelle R. Padget, Cailyn Lee, Zhen Su, Jacques Moisan, Madan Katragadda, Jeffrey Schlom, James L. Gulley, Andrew Bayliffe, James W. Hodge

**Affiliations:** 1 Center for Immuno-Oncology, Center for Cancer Research, National Cancer Institute, National Institutes of Health, Bethesda, MD, United States; 2 Marengo Therapeutics, Cambridge, MA, United States

**Keywords:** breast cancer, combination immune therapies, docetaxel, prostate cancer, TcE (T-cell engager)

## Abstract

**Background:**

STAR0602 is a selective bifunctional T cell agonist targeting Vβ6/Vβ10 T-cell receptors fused to interleukin-2, with emerging clinical activity in anti-PDL1–resistant tumors. Its murine surrogate, mSTAR1302, expands Vβ13 T cells and mediates antitumor activity. Docetaxel, beyond its cytotoxic effects, induces immunogenic modulation of tumor cells. We hypothesized that docetaxel-driven tumor sensitization would enhance susceptibility to immune-mediated killing, while mSTAR1302 would expand functional T cell subsets, resulting in coordinated antitumor responses.

**Methods:**

The therapeutic efficacy and mechanism of action of docetaxel and mSTAR1302 combination therapy were evaluated in 4T1 triple-negative breast cancer and TRAMP-C2 prostate cancer models. Immune profiling, functional assays, and CRISPR-mediated gene knockdown were used to define mechanisms of response.

**Results:**

Combination therapy significantly reduced tumor burden and improved survival compared to monotherapies in both models. Docetaxel induced immunogenic modulation characterized by upregulation of MHCI, FAS, and TRAIL-R2, enhancing tumor susceptibility to immune-mediated lysis. Mechanistically, TRAIL-R2 expression contributed substantially to antitumor activity, as knockdown attenuated therapeutic efficacy. mSTAR1302 expanded Vβ13+ CD4+ and CD8+ tumor-infiltrating lymphocytes and promoted antigen-specific T cell responses. Depletion studies demonstrated that CD4+ T, CD8+ T, and NK cells collectively mediated the antitumor effects of combination therapy.

**Conclusions:**

These findings identify a mechanistic axis in which docetaxel-induced tumor sensitization via TRAIL-R2 cooperates with Vβ-targeted T cell expansion to drive coordinated immune-mediated tumor regression. This work supports the clinical evaluation of STAR0602 in combination with chemotherapy and highlights TRAIL-R2–mediated tumor sensitization as a mechanistically defined strategy to enhance immunotherapy efficacy in immune-excluded tumors.

## 1 Introduction

Breast cancer is the second most diagnosed cancer worldwide and the most common in women, with more than 310,000 new cases diagnosed in 2024 within the United States (U.S.) alone (1) and 2.2 million globally (2). Triple negative breast cancer (TNBC) or breast cancers that lack expression of receptors for estrogen and progesterone with low expression of human epidermal growth factor receptor 2 (HER2) are the most malignant type of breast cancer, accounting for 10-15% of all diagnosed cases (2, 3). While the overall survival rate for breast cancer is approximately 90%, those diagnosed with TNBC have a decreased risk of survival due to a lack of targetable markers, a higher grade of disease, and diagnosis at a later stage (3). Similarly, prostate cancer is the most commonly diagnosed cancer in men in the U.S., with more than 299,000 new cases and an estimated 35,000 deaths in 2024 (1). Mortality is highly associated with metastatic disease in breast and prostate cancer (1).

Docetaxel is a chemotherapeutic agent in the taxane family that is used as the standard of care for many cancers, including breast, prostate, head and neck, and non-small cell lung cancers (4). The drug’s mechanism of action involves binding to tubulin and stabilizing microtubules, ultimately disrupting the mitotic process and leading to cell cycle arrest and cell death (5). In breast cancer, docetaxel is used to treat locally advanced and metastatic breast cancers following failed chemotherapy regimens (6, 7). In prostate cancer, it is used to treat metastatic castration-resistant cancers in combination with prednisone (7, 8). Although docetaxel has been known to improve survival across many cancer indications, chemoresistance and relapse remain issues following its use (9–11). In recent years, docetaxel has been studied in combination with various immunotherapeutic agents, including checkpoint inhibitors, adoptive T-cell therapies, tumor-targeted superantigens, and recombinant vaccines. It has shown increased efficacy with combination strategies compared with single agents (12–15). Docetaxel’s ability to synergize with such a wide array of immunotherapeutic agents is, in part, attributed to its ability to induce immunogenic modulation, a phenomenon in which sublethal doses of specific chemotherapeutic agents or radiation induce phenotypic changes within tumor cells, making the tumor more susceptible and targetable for immune system attack (12, 16–18).

STAR0602 is a bifunctional therapeutic molecule designed to selectively target, activate, and expand a subset of αβ T cells predominantly expressing the Vβ6 chain of the T-cell receptor (TCR). RNA sequencing analysis of tumor-infiltrating lymphocytes (TILs) across several cancers has revealed that Vβ6 TCR-expressing T cells are among the most frequent Vβ-chain T-cell subsets and are common to all solid tumors (19). STAR0602 was therefore designed as an antibody-fusion molecule comprising a Fab fragment targeting the Vβ6 TCR and native human interleukin-2 (IL-2), both fused to a human IgG1 Fc region. With this molecule design, STAR0602 conditionally agonizes both the TCR and IL-2 receptors (IL-2R) in *cis* on the same Vβ6 T cell. The ongoing STARt-001 Phase 1/2 monotherapy trial of intravenously infused STAR0602 has reported clinically meaningful antitumor activity in anti-programmed death ligand 1 (PD-L1)–resistant tumors, with an acceptable tolerability and safety profile in patients with advanced antigen-rich solid tumors (NCT05592626) (20).

mSTAR1302 is the murine surrogate molecule of STAR0602, which targets and activates Vβ13 TCRs in mice. Preclinical studies confirm the expansion of Vβ13-expressing CD4^+^ and CD8^+^ memory-like effector T cells following mSTAR1302 treatment and significant antitumor activity as monotherapy across multiple mouse tumor models, compared with anti-programmed cell death protein 1 (PD-1) therapy in checkpoint inhibition–resistant cancer models (21). In a recent study, mSTAR1302 was used in combination with standard-of-care cisplatin and anti-PD1 in head and neck squamous cell carcinoma (HNSCC), resulting in increased antitumor activity and survival compared to monotherapies in MOC1 and MOC2 tumor models (22).

Here, we demonstrate, for the first time, the antitumor effects and survival benefits of docetaxel in combination with the novel bifunctional T-cell activator, mSTAR1302, in models of triple-negative breast and androgen-independent prostate cancer. In the 4T1 breast cancer model, lung metastases were reduced, accompanied by increased infiltration of cytotoxic lymphocytes and upregulation of death receptor expression within the tumor and its microenvironment. Taken together, these data support further clinical evaluation of docetaxel and STAR0602 in breast and prostate cancers. Importantly, this study also seeks to define the mechanistic basis of this combination, specifically whether chemotherapy-induced tumor sensitization via death receptor signaling can be functionally coupled with selective Vβ-targeted T-cell expansion to enhance immune-mediated tumor clearance.

## 2 Materials and methods

### 2.1 Cell lines, animals, and reagents

The murine TNBC cell line 4T1 and androgen-independent prostate cell line TRAMP-C2 were obtained from the American Type Culture Collection (ATCC; Manassas, VA, USA) and cultured according to ATCC recommendations. Cells were used at low passage numbers (<20) and were confirmed to be *Mycoplasma*-free. Wild-type (WT) female Balb/c mice and WT male C57BL/6 mice were obtained from Charles River Laboratory (Wilmington, MA, USA) or bred in-house at the National Institutes of Health (NIH; Bethesda, MD, USA). All mice used in the study were housed and maintained at the NIH in microisolator cages in pathogen-free conditions. Tumor necrosis factor-related apoptosis-inducing ligand receptor (TRAIL-R2) clustered regularly interspaced short palindromic repeats (CRISPR) knockouts (KOs) in 4T1 and TRAMP-C2 cells were generated using recombinant TrueCut Cas 9 protein version 2 and TrueGuide synthetic guide RNA targeting murine TRAIL-R2 (ID number CRISPR514558_SGM; targeting DNA sequence: CTATATAGAAGTCTGCGCTT) using the Lipofectamine CRISPRMAX transfection reagent, following the manufacturer’s recommendations (Thermo Fisher Scientific; Waltham, MA, USA). TRAIL-R2 knockout CRISPR cells were stained for TRAIL-R2 expression using PE-conjugated mouse TRAIL-R2 antibody (clone MD5-1; BioLegend, San Diego, CA, USA) and single-cell sorted using MA900 cell sorter with a 100µm sorting chip (Sony Biotechnology; San Jose, CA, USA). Clones were selected for *in vivo* experiments based on tumor growth rates similar to those of the parental tumor model. Docetaxel (Winthrop Sanofi Company; Gentilly, Paris, France) was obtained from the NIH pharmacy. mSTAR1302 was provided by Marengo Therapeutics (Cambridge, MA, USA) through a Cooperative Research and Development Agreement (CRADA) with the National Cancer Institute (NCI), NIH (Bethesda, MD, USA).

### 2.2 Tumor lysis assays

Spleens were excised from untreated non-tumor–bearing Balb/c mice and manually dissociated. ACK (Ammonium Chloride Potassium) Lysing Buffer (Quality Biological; Gaithersburg, MD, USA) was used to remove red blood cells (RBCs). Natural killer (NK) cells were isolated from the splenocytes using a magnetic CD49b^+^ positive selection kit (Miltenyi Biotec; Bergisch Gladbach, Germany) and cultured overnight in RPMI medium at 37 °C. At the time of assay, 4T1 tumor cells were exposed to docetaxel (250 ng/mL) for 48 hours, harvested, and stained with 10 µM calcein AM (Invitrogen, Waltham, MA, USA). Immediately thereafter, tumor cells and NK cells were co-cultured in a 384-well flat-bottom plate on the IncuCyte^®^ SX5 Live-Cell Analysis System (Sartorius; Göttingen, Germany) in an effector-to-target (E:T) ratio of 25:1. Cell lysis was measured via a decrease in total area of fluorescence normalized to the tumor cells only control.

To examine the role of docetaxel treatment on TRAIL-R1/2 and FAS (CD95)–mediated lysis, 4T1 and TRAMP-C2 tumor cells were treated with docetaxel (250ng/mL) or no drug for 48 hours. Tumor cells were plated at 15,000 cells/well in a Real-Time Cell Analysis (RTCA) microplate on the xCELLigence RTCA MP (Agilent; Santa Clara, CA, USA) and allowed to adhere overnight. After 24 hours, solubilized murine TRAIL protein at 1500 ng/mL (Enzo Life Sciences; Farmingdale, NY, USA) or anti-FAS (clone Jo2; BD Biosciences; Franklin Lakes, NJ, USA), with and without Protein G (10 µg/mL), was added to the tumor cells. Impedance was measured every 15 minutes and used to determine cell lysis.

### 2.3 *In vivo* experiments

All animal studies were conducted in accordance with the NCI-Bethesda Animal Care and Use Committee–approved animal protocol CIO-002 and utilizing ARRIVE reporting guidelines. For each experiment, mice were either untreated, treated with docetaxel or mSTAR1302 as a monotherapy, or treated with docetaxel and mSTAR1302 in combination. Untreated mice were used as the control. A single animal is considered an experimental unit in all studies. In the depletion studies, mice were either untreated or treated with the combination therapy as controls. The number of mice per group, total number of mice per experiment, and total number of mice used in data analysis are depicted in the Figure legends for each experiment. Sample size was determined from previous studies. Balb/c mice were randomized within each group according to tumor size. C57BL/6 mice were randomized by average tumor volume in each cage. Both strategies were implemented to minimize differences in average tumor volumes across groups. To minimize potential confounders, mice of the same strain, genetic background, and age were used, and mice from each experiment were housed in the same building and location. No criteria were established to include or exclude animals once the study began; however, only mice with tumor volumes within a specified range were used in each study, excluding those outside that range. Mice were also excluded from the study if it was determined that the tumors were implanted inappropriately (i.e., tumors were implanted intramuscularly instead of subcutaneously (s.c.)). To assess the efficacy and safety of the treatment, the tumor volumes, weights, and lung metastases, when applicable, were measured. The study was not blinded, and all researchers were aware of group assignments during the experiments, outcome assessments, and data analysis. All drug treatments and depletions were administered in the animal facility, while all post-mortem tissue or blood collections were performed in the laboratory.

For the TNBC model, female Balb/c mice, aged 8–12 weeks, were implanted s.c. with 5 × 10^4^ 4T1 cells in the right mammary fat pads on day 0. Treatment began between days 9 and 11, when tumors reached approximately 40-70mm^3^. For the prostate model, C57bl/6 male mice (8–12 weeks old) were implanted (s.c.) on day 0 in the right flank with 2.5 × 10^6^ TRAMP-C2 cells mixed 1:1 with Matrigel (Corning; Corning, NY, USA). Treatment began on day 22 when tumors were between 70–100 mm^3^. Tumor volumes were calculated using the following formula: (length x width ^2^)/2. In both models, mSTAR1302 was administered intraperitoneally (i.p.) at 1 mg/kg, with a total of three doses, each given 1 week apart. Docetaxel treatment commenced concurrently with the second mSTAR1302 dose and was administered i.p. at 500 µg, equivalent to the clinical use of 75–100 mg/m², every other day for a total of three doses. In the 4T1 model, an alternative treatment schedule was also explored, starting with docetaxel treatments (500 µg, i.p.) on days 10, 12, and 14, followed by mSTAR1302 treatments (1 mg/kg, i.p.) on days 17, 24, and 31.

To assess the activation of Vβ13+ T cells following mSTAR1302 treatment, non-tumor–bearing female Balb/c mice (8–12 weeks old) were given a single dose of mSTAR1302 (1 mg/kg, i.p.). Spleens were excised between days 0 and 9 post-injection with mSTAR1302. To understand how docetaxel impacts mSTAR1302-dependent Vβ13 activation, female non-tumor–bearing Balb/c mice (8–12 weeks old) were either treated with docetaxel (days 0, 2, 4) followed by a single dose of mSTAR1302 (day 7) or treated with mSTAR1302 on days 0 and 7 with docetaxel on days 7, 9, and 11. Spleens were excised between days 0 and 16 and analyzed by flow cytometry.

For depletion studies, female Balb/c mice were implanted with 4T1 cells in the mammary fat pad (5×10^4^ cells, s.c.). All groups, except the untreated group, received mSTAR1302 (1 mg/kg, i.p.) on days 9, 16, and 23, and docetaxel (500 µg, i.p.) on days 9, 11, and 13. Anti-CD4 (clone GK1.5; 100 µg, i.p.; BioXcell, Lebanon, NH, USA) and anti-CD8 (clone 2.43; 100 µg, i.p.; BioXcell) depleting antibodies were administered on days 6, 7, and 8, and every 7 days thereafter. NK depletion was achieved using anti-Asialo-GM1 (Poly21460; Biolegend) administered at 25 µL in 100 µL of PBS i.p. on days 6 and 8, and every 7 days thereafter. Anti-interferon (IFN)-γ (XMG1.2; 100 µg, i.p., BioXcell) was given three times per week, 2 days before mSTAR1302 treatment, the same day as mSTAR1302 treatment, and 2 days following mSTAR1302 treatment (BioXcell). In all studies, mice were monitored weekly and euthanized when an ethical limit was approached, which included a tumor volume of 2,000 mm³, any tumor dimension of 20 mm or greater, a loss of 20% or more in body weight, or tumor ulceration covering 50% of the surface area. Mice were euthanized by carbon dioxide (CO_2_) inhalation in accordance with institutional animal care and use committee (IACUC) guidelines.

### 2.4 Flow cytometry

Cells were harvested and processed into single-cell suspensions, washed, and stained.

Live/Dead stain in fixable blue (Thermo Fisher Scientific) was used to differentiate live and dead cells. Anti-CD16/CD32 (Clone 93; Invitrogen) Fc-blocking antibody was used to prevent nonspecific binding. The following murine fluorophore conjugated primary antibodies were used: H-2k^b^-FITC (clone AF6-88.5), H2k^d^-FITC (SF1-1.1), CD3-APC-Cy7 (17A2), CD8-PerCP-Cy.5.5 (54-6.7), Vβ8.1-PE (KJ16133.18), CD49b-BV786 (HMa2), CD11b (PerCP-Cy5.5) from BD Biosciences (Franklin Lakes, NJ, USA), FAS(CD95)-BV605 (SA367H8), CD4-BV605 (RM4-5), Vβ8.1-FITC (KJ16133.18), F4/80-BV605 (BMB), CD38-BV421 (90), and CD207-PE-Cy7 (C06802) from BioLegend, TRAIL-R2 (CD262)-PE (MD5-1) from Invitrogen, and CD45-BUV737 (30-F11) from Thermo Fisher. Sample data were acquired on a BD LSRFortessa using FACSDiva software and analyzed with FlowJo V10.10.0 (BD Biosciences).

### 2.5 OPAL immunofluorescence

4T1 tumor-bearing Balb/c mice were treated with no drug, docetaxel, and/or mSTAR1302. Tumors were surgically removed, fixed in Z-fix (Anatech; Battle Creek, MI, USA), and sent to VitroVivo Biotech (Gaithersburg, MD, USA) for paraffin embedding and sectioning. Slides were stained using the Opal 4-Color Manual IHC Kit from Akoya Biosciences (Marlborough, MA, USA), according to the manufacturer’s instructions. The following antibodies were used: anti-CD8 (ab217344), anti-CD4 (ab183685), and TRAIL-R2 (ab8416) from Abcam (Waltham, MA, USA), and FAS (Pa5-115214) from Invitrogen. Secondary-only staining was used as a negative control. Slides were scanned using the Azio Scan Z1 with Zen software (Zeiss; Oberkochen, Germany). Samples were analyzed using five representative images per tumor per group, and the number of nuclei per region was quantified. Cell counting was automated and performed using the Otsu thresholding method.

### 2.6 RNA analysis

Total RNA was extracted using the Qiagen RNeasy Mini Kit (Qiagen; Hilden, Germany) from tumor cells isolated from 4T1 tumor-bearing female Balb/c mice on day 23, following treatment with no drug, docetaxel, mSTAR1302, or combination. RNA data were analyzed by the Genomics Laboratory at Frederick National Laboratory for Cancer Research using the NanoString Technologies nCounter Murine PanCancer Immune Profiling panel. nSolver V4.0 analysis software (NanoString Technologies; Seattle, WA, USA) was used to analyze and normalize gene expression data. Ingenuity Pathway Analysis V01-23-01 (Qiagen; Benio, The Netherlands) was used for pathway analyses. Cell Type Profiling Analysis was conducted using ROSALIND (NanoString Technologies).

### 2.7 ELISPOT

On day 23, the spleens of 4T1 tumor-bearing female Balb/c mice treated with docetaxel, mSTAR1302, or a combination of both, or no drugs, and were surgically removed, manually processed, and lysed to remove RBCs. Splenocytes were plated onto ELISPOT plates previously coated with IFN-γ–specific capture antibody (551083; BD Biosciences) overnight at 4 °C and then blocked with cell culture medium at room temperature for 2 hours. The splenocytes were stimulated with AH1 peptide (SPSYVYHQF; GenScript, Piscataway, NY, USA) or β-gal (TPHPARIGL; CPC Scientific, Rocklin, CA, USA) or stimulated with anti-CD3 (clone 145-2C11; Invitrogen) and anti-CD28 (Clone 37.51; Biolegend) overnight at 37 °C with 5% CO_2._ Plates were developed with detection antibody from BD ELISPOT Mouse IFN-y ELISPOT Set and BD ELISPOT AEC Substrate Set (BD Biosciences). ELISPOT plates were pictured and quantified using the CTL ImmunoSpot Analyzer (Immunospot; Cleveland, OH, USA).

### 2.8 Statistical analysis

Statistical analyses were performed using GraphPad Prism. For comparisons involving more than two groups, one-way or two-way ANOVA with Tukey’s *post hoc* test was used to account for multiple comparisons within each experiment. Reported p-values were not adjusted for false discovery rate (FDR) across independent experiments and should be interpreted in the context of hypothesis-driven analyses. The log-rank (Mantel-Cox) test was used to analyze the survival curve data. Fisher’s exact test was used for RNA pathway analyses. The non-parametric Kolmogorov-Smirnov (KS) test was used to compare distributions between the histograms comparing two groups using FlowJo V10.10.0 (BD Biosciences). All error bars represent mean ± SEM.

## 3 Results

### 3.1 Combination therapy with docetaxel and mSTAR1302 elicits antitumor effects in triple-negative breast cancer (4T1) and androgen-independent prostate cancer (TRAMP-C2) mouse models

**Figure 1:**
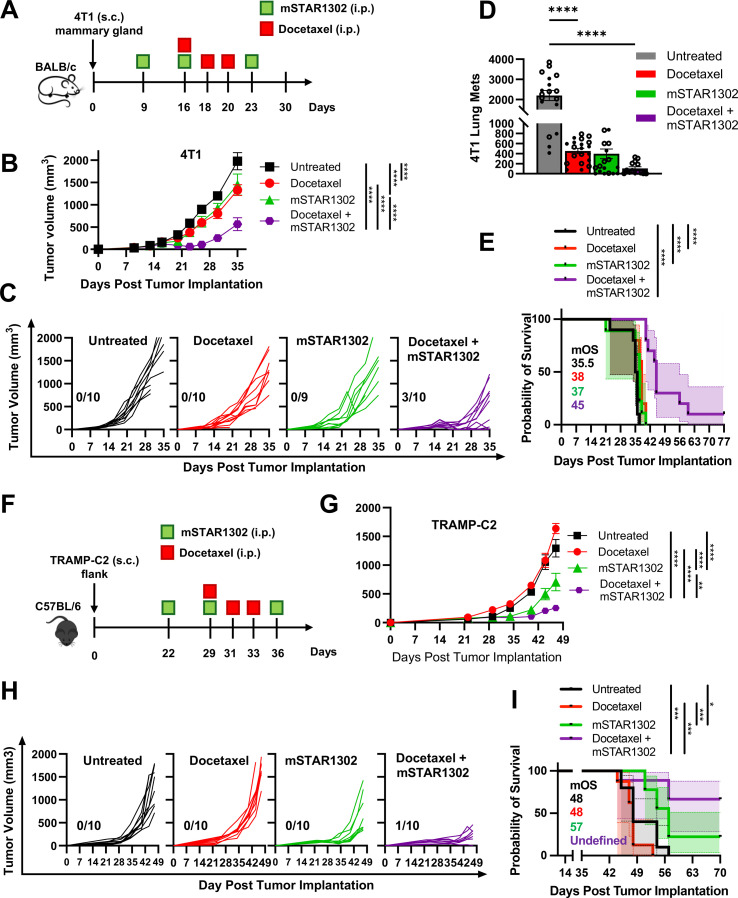
Combination therapy with docetaxel and mSTAR1302 elicits antitumor effects in triple negative breast cancer (4T1) and androgen-independent prostate cancer (TRAMP-C2) mouse models. **(A–E)** 8–12-week-old female Balb/c mice were inoculated subcutaneously with 5x10^4^ 4T1 in the mammary fat pad (n=8–10 per group). **(A)** pictogram depicts experimental design. When the tumors reached 40-70mm^3^, mice were administered 3 doses of mSTAR1302 (1 mg/kg, i.p.) once per week on days 9, 16, and 23 and 3 doses of docetaxel (500 µg, i.p.), every other day, 7 days post-initial mSTAR1302 treatment, on days 16, 18, and 20. Primary tumors were measured in terms of mean **(B)** and individual **(C)** tumor volumes, as depicted. Inset numbers are tumor-free mice. In two additional cohorts, to assess metastatic growth, lungs were removed from 4T1 tumor-bearing mice 28 days post-tumor inoculation following the same treatment schedule. Lungs were dissociated, cultured in medium (2x FBS with 6-thioguanine) and incubated at 37 °C with 5% CO_2_ for 12 days. **(D)** a meta-analysis from two separate studies is depicted with closed and opened circles differentiating data collected from each study. Cell colonies representing lung metastases were stained with 0.05% methylene blue and counted for each treatment group (n=14–20 per group total). Survival **(E)** was measured over time with inset numbers depicting median overall survival (mOS) and shaded bands depicting 95% confidence intervals. **(F–I)** 8–12-week-old male C57bl/6 mice were inoculated subcutaneously with 2.5x10^6^ TRAMP-C2, on the right flank (n=8–10 per group). **(F)** pictogram depicts experimental design. Mice were administered mSTAR1302 (1 mg/kg, i.p.) when tumors reached 70–100mm^3^ on days 22, 29, and 36, and docetaxel (500 µg, i.p.) on days 29, 31, and 33. Tumors were measured with mean **(G)** and individual **(H)** tumor volumes depicted. Inset numbers are tumor-free mice. Survival **(I)** was measured over time with mOS and 95% confidence intervals (shaded bands) depicted. Statistical tests: tumor growth: two-way ANOVA with Tukey’s *post hoc* test; comparison between groups: one-way ANOVA with Tukey’s *post hoc* test; survival; Mantel-Cox test. Error bars, SEM. *p*<*0.05, **p*<*0.01, ***p*<*0.001, ****p*<*0.0001. ANOVA, analysis of variance; FBS, fetal bovine serum; i.p., intraperitoneal; mets, metastases; s.c., subcutaneously.

To determine the efficacy of docetaxel and mSTAR1302 treatment in a TNBC model, female Balb/c mice were inoculated with 4T1 tumors and treated as depicted in **Figure 1A**. Monotherapy with docetaxel (p*<*0.0001) reduced tumor volume compared with untreated mice. Mice treated with mSTAR1302 monotherapy (p*<*0.0001) also showed a decrease in tumor volume compared to the untreated cohort. Mice treated with the docetaxel and mSTAR1302 combination, however, exhibited significant antitumor activity compared to untreated mice (p*<*0.0001) and mice treated with docetaxel (p*<*0.0001) or mSTAR1302 (p*<*0.0001) as single modalities (**Figures 1B, C**). Furthermore, only the combination treatment with docetaxel and mSTAR1302 resulted in 33% (3 out of 10) primary tumor-free mice on day 35 of the study (**Figure 1C****);** however, the tumors recurred weeks after the end of the treatment regimen. Additionally, the combination treatment of docetaxel and mSTAR1302 was well tolerated in mice, with no significant changes in body weight. In all groups, following the final administration of either docetaxel or mSTAR1302, the average body weight of the mice was between 22 and 23 grams, compared to an average initial body weight of 23 grams for each treatment group (**Supplementary Figure 1**).

To independently assess the impact on metastatic progression, lungs from 4T1 tumor-bearing mice were harvested and analyzed for metastatic burden. Combination-treated mice exhibited a marked reduction in lung metastases compared to all other groups (**Figure 1D****),** indicating that the therapy also limits metastatic dissemination and/or outgrowth. Docetaxel-treated mice showed a 79% (5-fold) reduction in lung metastases compared to untreated mice (p*<*0.0001), while mSTAR1302-treated mice exhibited an 82% reduction (p*<*0.0001). Within the cohort treated with the combination therapy, lung metastases were reduced, most notably by 95% (20-fold difference) (p*<*0.0001) (**Figure 1D**). Conversely, only mice treated with a combination of docetaxel and mSTAR1302 showed a survival benefit compared to untreated mice (hazard ratio (HR) 0.2199, 95% confidence interval (CI) 0.07091-0.6821, p<0.0001), docetaxel-treated mice (HR 0.2422, 95% CI 0.08063-0.7277, p<0.0001), or mSTAR1302-treated mice (HR 0.2247, 95% CI 0.06810-0.7412, p<0.0001) (**Figure 1E**).

To test whether the antitumor activity observed with the combination therapy of docetaxel and mSTAR1302 could be validated in a second tumor model, TRAMP-C2, an androgen-independent prostate cancer cell line, was implanted into the right flank of male C57BL/6 mice. Treatment commenced on day 22, as outlined in the treatment schedule depicted in **Figure 1F**. No therapeutic benefit was observed in docetaxel-treated mice compared to untreated mice. mSTAR1302 monotherapy, however, resulted in a significant increase in antitumor activity (p*<*0.0001) compared to untreated mice. The best tumor control was observed in combination-treated mice compared to untreated mice (p*<*0.0001), docetaxel-treated mice (p*<*0.0001), and mSTAR1302-treated mice (p*<*0.01) (**Figures 1G, H**). All treatment regimens were well tolerated, with mice losing no more than 15% of their initial body weight across all groups (**Supplementary Figure 1**). In the TRAMP-C2 model, combination-treated C57BL/6 mice showed a moderate reduction in body weight, but weight loss did not exceed 15% in any treatment group and remained below the prespecified humane endpoint of ≥20% body-weight loss. No treatment-related mortality was observed. Because these studies used a single docetaxel dose level, dose-dependence of this effect was not formally assessed. Docetaxel-treated mice had a median overall survival (mOS) of 48 days, with no significant difference in survival compared to untreated mice, with hazard ratio (HR) of 1.829 (95% confidence interval (CI) 0.6700-4.991, p<0.0827). Compared to control, the mSTAR1302-treated mice had an mOS of 57 days, suggesting improved survival compared with controls, although confidence intervals remained broad (HR 0.3853, 95% CI 0.1427-1.040, p=0.0137). The docetaxel and mSTAR1302 combination treatment provided the most significant survival benefits (HR 0.1857, 95% CI 0.06068-0.5684, p=0.0009), with 66% (6 out of 9) of mice surviving to day 70 of the study (**Figure 1I**). These data, taken together, confirm the efficacy of docetaxel and mSTAR1302 co-treatment in inducing tumor regression and improving survival across multiple tumor models compared with either treatment modality alone.

### 3.2 Schedule and timing of docetaxel and mSTAR1302 drug administration impacts Vβ13 expansion and antitumor activity

The therapeutic efficacy and toxicity of drugs can vary widely depending on scheduling and dosage. This is particularly true in this study, where mSTAR1302 was shown to rapidly expand T cells, while docetaxel, a chemotherapeutic, targeted rapidly tumor dividing cells. To investigate the optimal timing for docetaxel administration when combined with mSTAR1302, the spleens of non-tumor-bearing female Balb/c mice were collected and analyzed for the frequency of Vβ13-positive T cells following a single dose of mSTAR1302 over time. CD4^+^ Vβ13–expressing T cells reached their apogee of activation 4 days following a single dose of mSTAR1302 and more than doubled compared to baseline at day 0 (**Supplementary Figure 2A**). CD8^+^ Vβ13–expressing T cells similarly peaked in expression 4 days post-mSTAR1302 administration and increased 7-fold compared to baseline (**Supplementary Figure 2A**).

Additionally, we investigated whether the timing of docetaxel administration relative to mSTAR1302 affected the activation, expansion, and phenotype of Vβ13 T cells. In one cohort, mice were treated with mSTAR1302 (day 0) followed by a second dose of mSTAR1302 (day 7) in conjunction with docetaxel (days 7, 9, 11) (**Supplementary Figure 2B**). The second cohort received treatment with docetaxel (days 0, 2, 4) followed by a dose of mSTAR1302 (day 7) (**Supplementary Figure 2C**). Mice treated with mSTAR1302 at the start of the regimen showed greater expansion of Vβ13+ T cells at earlier time points than mice that received docetaxel first. Furthermore, when mSTAR1302 was administered first, the expansion of Vβ13^+^ T cells following docetaxel treatment and a second dose of mSTAR1302 was still observed. However, mice treated with docetaxel first showed a slower expansion of Vβ13^+^ T cells following mSTAR1302 administration (**Supplementary Figures 2B, C**).

The impact of the sequence of drug administration on tumor volume was assessed in the 4T1 model. Two different treatment schedules were investigated: treatment schedule 1 started with mSTAR1302, while treatment schedule 2 started with docetaxel (**Supplementary Figures 2D, E**). In Schedule 1, mSTAR1302 treatment was administered on days 10, 17, and 24, with docetaxel treatments on days 17, 19, and 21 (**Supplementary Figure 2D**). Schedule 2 began with docetaxel treatments on days 10, 12, and 14, followed by mSTAR1302 on days 17, 24, and 31 (**Supplementary Figure 2E**). Overall, the combination of docetaxel and mSTAR1302 treatment using Schedule 1 had the most significant antitumor effect, resulting in a 75% reduction in mean tumor volume compared with the control. Meanwhile, combination therapy using Schedule 2 resulted only in a 50% tumor size reduction when compared to control (**Supplementary Figures 2D, E**). In Schedule 1, docetaxel (p*<*0.0001) and mSTAR1302 (p*<*0.0001) showed significant monotherapy activity compared to untreated mice. More importantly, combination treatment showed a marked decrease in tumor volumes compared to the untreated group (p*<*0.0001), the docetaxel-treated group (p*<*0.0001), and the mSTAR1302-treated group (p*<*0.01). Schedule 2 also exhibited significant antitumor activity with combination treatment compared to untreated mice (p*<*0.0001) and mice treated with mSTAR1302 monotherapy (p*<*0.0001). However, no significant differences were observed between the cohorts receiving docetaxel and the combination in Schedule 2 (**Supplementary Figure 2E**). These data suggest that treatment with mSTAR1302 before docetaxel may increase therapeutic benefit. Based on these data, schedule 1 was utilized for all remaining studies.

### 3.3 Docetaxel and mSTAR1302 combination induces upregulation of the death receptors TRAIL-R2 and FAS in 4T1 and TRAMP-C2 cells

**Figure 2:**
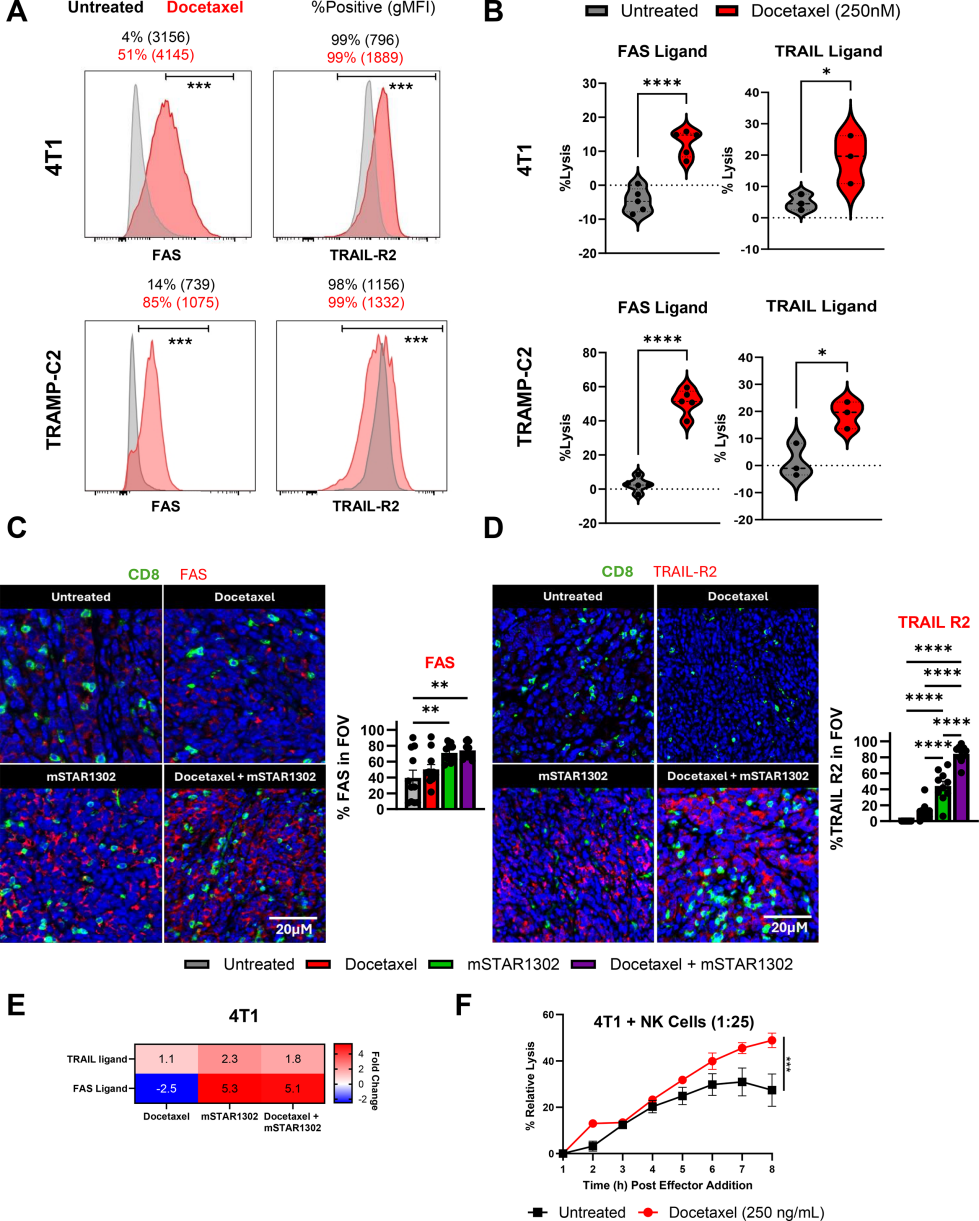
Docetaxel induces upregulation of death receptors TRAIL-R2 and FAS in 4T1 and TRAMP-C2 cells. **(A)** 4T1 and TRAMP-C2 cells were treated *in vitro* with either no drug or docetaxel (250 ng/mL) for 48 hours and analyzed for surface expression of FAS and TRAIL-R2 via flow cytometry. Histograms indicating frequency and gMFI are shown. Experiment repeated twice with similar results. **(B)** 4T1 and TRAMP-C2 cells were treated as described in **Figure 1A** and then co-cultured with TRAIL ligand or FAS ligand and protein G and measured for relative cell lysis compared to cells alone. **(C, D)** tumors harvested on day 28, after treatment as described in **Figure 1A**, were fixed for multiplex immunofluorescence staining and quantified for CD8 (green) and TRAIL-R2 or FAS (red) expression. **(E)** differential gene expression was interrogated using NanoString murine PanCancer immune profiling panel on tumors harvested on day 23 from 4T1 tumor-bearing Balb/c female mice, following treatment as previously described. Fold change compared to untreated tumors reported for TRAIL and FAS ligands. **(F)** 4T1 cells were exposed to docetaxel (250 ng/mL) for 48 hours and co-cultured with NK cells isolated from tumor-free Balb/c mice (E:T = 25:1) and monitored for relative NK-specific lysis of tumor cells for up to 8 hours post-effector addition. Statistical tests: comparison between groups: One-way ANOVA with Tukey’s *post hoc* test. comparison between two groups: unpaired T-test. Error bars, SEM. *p*<*0.05, **p*<*0.01, ***p*<*0.001, ****p*<*0.0001. ANOVA, analysis of variance; FoV, field of view; gMFI, geometric mean fluorescence intensity; NK, natural killer.

Previous studies have shown that docetaxel can induce immunogenic changes in a variety of tumor cell types, thereby making tumors more susceptible to immune targeting and other treatment modalities (12, 16, 23). To understand the immunogenic modulation potential of docetaxel in 4T1 and TRAMP-C2 tumor cell lines, tumor cells were treated with docetaxel for 48 hours *in vitro* and then analyzed by flow cytometry. Docetaxel treatment mediated upregulation of the apoptotic signaling molecules TRAIL-R2 and FAS in both 4T1 and TRAMP-C2 cell lines. In 4T1 tumor cells, the expression of FAS increased from 4% (mean fluorescence intensity (MFI) of 3156) to 51% (4145), while the MFI of TRAIL-R2 increased from 796 to 1889 following docetaxel treatment. FAS expression in TRAMP-C2 increased from 14% (739) to 85% (1075), while TRAIL-R2 increased only marginally with an MFI of 1156 to 1332 post-docetaxel treatment (**Figure 2A**). In 4T1, the expression of H-2k^d^-FITC, the major histocompatibility class I (MHCI) cell surface marker, increased from 36% (2276) to 84% (4272) with docetaxel treatment. Similarly, H-2k^b^-FITC expression increased from 9% (747) to 81% (1049) in TRAMP-C2 cells following docetaxel treatment (**Supplementary Figure 3**). These findings suggest that docetaxel-induced upregulation of death receptors, particularly TRAIL-R2, may represent a key sensitization mechanism that enhances susceptibility to immune-mediated cytotoxicity.

We next assessed the functional role of TRAIL-R2 and FAS on the immunomodulatory effects of docetaxel. This was done by assessing the functional ability of tumor cells with upregulated TRAIL-R2 or FAS to induce cell lysis when exposed to exogenous forms of their respective ligands. 4T1 and TRAMP-C2 cells were treated with docetaxel for 48 hours. The addition of TRAIL ligand onto cells treated with docetaxel caused a significant increase in relative cell lysis in 4T1 (p*<*0.05) and TRAMP-C2 (p*<*0.05) cell lines (**Figure 2B**). Similarly, the addition of FAS ligand to cells treated with docetaxel increased relative lysis in 4T1 and TRAMP-C2 tumor cells (**Figure 2B**).

Next, to assess whether docetaxel-induced upregulation of TRAIL-R2 and FAS expression is potentiated when combined with mSTAR1302 *in vivo*, 4T1 tumor-bearing Balb/c mice were treated as described in **Figure 1A**. Their tumors were excised on day 28, 8 days after the last docetaxel treatment and 5 days after the previous mSTAR1302 treatment. Tumor sections were stained for CD8 and TRAIL-R2 or FAS. Mice treated with mSTAR1302 monotherapy exhibited an increase in TRAIL-R2 expression (p*<*0.0001) compared to the untreated and docetaxel-treated groups. Mice receiving mSTAR1302 monotherapy (p*<*0.001) or a combination of docetaxel and mSTAR1302 (p*<*0.001) had an increase in expression of FAS compared to untreated mice (**Figure 2C**). Mice treated with both docetaxel and mSTAR1302 also had a significant upregulation in TRAIL-R2 compared to untreated (p*<*0.0001), mSTAR1302 monotherapy (p*<*0.0001), and docetaxel monotherapy (p*<*0.0001) groups (**Figure 2D**). Infiltration of CD8^+^ T cells also increased in tumors co-treated with docetaxel and mSTAR1302 (**Figures 2C, D**). Notably, RNA from tumors excised on day 22 also had an upregulation in the expression of TRAIL and FAS ligands in mice treated with mSTAR1302 or the combination of docetaxel and mSTAR1302 (**Figure 2E**). Lastly, we assessed the functional consequences of phenotypic changes mediated by docetaxel in NK-mediated cell killing. To achieve this, 4T1 cells were treated with docetaxel and co-cultured with NK cells isolated from the spleens of non-tumor–bearing Balb/c mice. There was a 27% increase in NK-mediated cell lysis following docetaxel treatment (p*<*0.001) in 4T1 tumor cells (**Figure 2F****).** Docetaxel treatment alone induced upregulation of TRAIL-R2 and FAS in tumor cells *in vitro*, indicating a tumor cell–intrinsic sensitization mechanism. In contrast, mSTAR1302 treatment *in vivo* was associated with increased expression of TRAIL and FAS ligand, consistent with immune-mediated activation. The combination of docetaxel and mSTAR1302 resulted in the highest levels of death receptor and ligand expression, supporting a cooperative mechanism between tumor sensitization and immune activation.

### 3.4 Antitumor activity of docetaxel and mSTAR1302 was attenuated in TRAIL-R2 knockdown tumor mouse models

**Figure 3:**
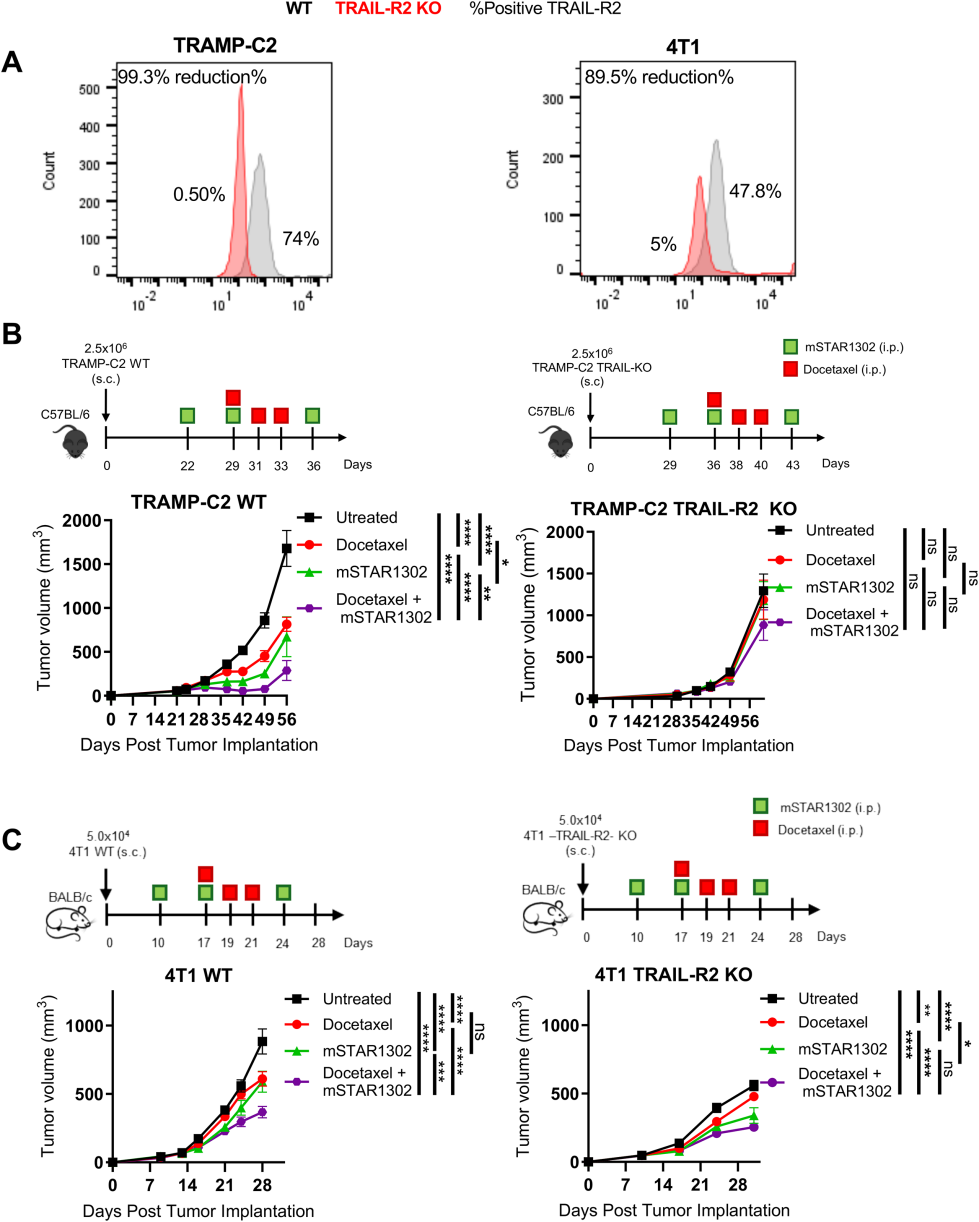
Antitumor activity of docetaxel and mSTAR1302 attenuated in TRAIL-R2 knockdown tumor mouse models. **(A)** TRAIL-R2 was knocked down via CRISPR in TRAMP-C2 and 4T1 cell lines. Flow cytometry was used to confirm expression in clones and WT cell lines. **(B)** 8–12-week-old male C57bl/6 mice were implanted with 2.5x10^6^ TRAMP-C2 (n=10–12 per group) or TRAMP-C2 TRAIL-KO (n=7–9 per group) cells in the right flank. Treatment schedules for each group are depicted. Mean tumor volumes graphed. **(C)** 8–12-week-old female Balb/c mice were implanted with 5x10^4^ 4T1 (n=18–20 per group) or 4T1 TRAIL KO cells (n=10) in the mammary fat pad. WT 4T1 mice from this study were assessed concurrently in the study from **Figure 7** and mean tumor volumes displayed in **Figure 7C** are the same tumor volumes displayed in **Figure 7B**. Statistical tests: tumor growth: two-way ANOVA with Tukey’s *post hoc* test. Error bars, SEM. *p*<*0.05, **p*<*0.01, ***p*<*0.001, ****p*<*0.0001. WT, wild type; KO, knockouts; CRISPR, clustered regularly interspaced short palindromic repeats; i.p., intraperitoneal; s.c., subcutaneously; ns, not significant.

Next, we wanted to assess the functional importance of TRAIL-R2 and FAS on the antitumor activity of docetaxel and mSTAR1302. Previously, we showed increased FAS and TRAIL-R2 expression in 4T1 tumors (**Figures 2C, D**) following combination treatment with docetaxel and mSTAR1302. To better understand the impact of FAS and TRAIL-R2 expression, CRISPR was performed on both 4T1 and TRAMP-C2 cell lines. TRAIL-R2 was successfully knocked down in both models, as confirmed by flow cytometry (**Figure 3A****).** C57BL/6 mice were inoculated with WT TRAMP-C2 or TRAMP-C2 TRAIL-R2 KO cells (**Figure 3B**) and Balb/c mice with WT 4T1 or 4T1 TRAIL-R2 KO cells (**Figure 3C**). Treatment schedules for each group are depicted in **Figures 3B, C**, with treatment starting in TRAMP-C2 TRAIL-KO on day 29, compared to day 22, when tumors reached their appropriate tumor volumes (70–100 mm^3^). Both monotherapy with mSTAR1302 and docetaxel co-treatment reduced tumor volumes in TRAMP-C2 WT tumors (**Figure 3B**). Differences in docetaxel monotherapy responses across experiments likely reflect variability in tumor growth kinetics and microenvironmental context in the TRAMP-C2 model; however, the consistent enhancement observed with combination therapy supports the robustness of the cooperative mechanism. Antitumor activity was completely abrogated in the TRAMP-C2 model in the absence of TRAIL-R2 expression (**Figure 3B**). The 4T1 TRAIL-R2 KO model exhibited partial but reduced sensitivity to docetaxel and mSTAR1302, as single agents or in combination, compared with the control (**Figure 3C**). The differential response observed between TRAMP-C2 and 4T1 models may, in part, reflect differences in TRAIL-R2 knockdown efficiency; however, additional factors, including tumor-intrinsic biology and potential redundancy in death receptor pathways, may also contribute. Collectively, these data identify TRAIL-R2 as a critical mediator of the antitumor activity observed with docetaxel and mSTAR1302 combination therapy, supporting a functional role for death receptor–dependent tumor sensitization in this therapeutic context.

### 3.5 Co-treatment of 4T1 tumors with docetaxel and mSTAR1302 enhances tumor-infiltrating lymphocytes

**Figure 4:**
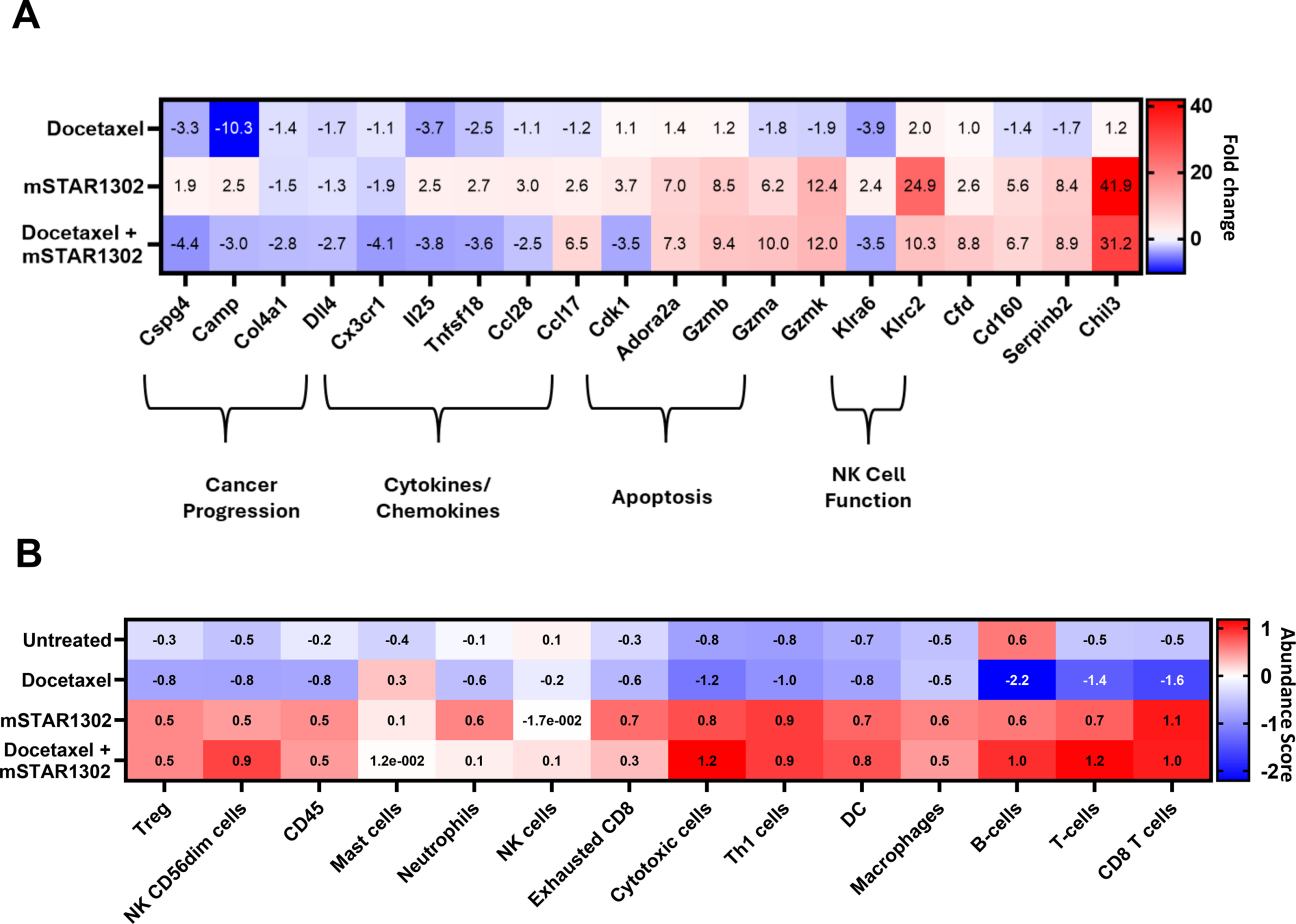
Co-treatment of 4T1 tumors with docetaxel and mSTAR1302 enhances tumor-infiltrating lymphocytes. RNA was isolated from tumors collected on day 23 from 4T1 tumor-bearing Balb/c female mice, following treatment, as previously described in **Figure 1A**. RNA analysis performed via NanoString murine PanCancer immune profiling panel. Heatmaps displaying **(A)** the top 10 differentially upregulated and downregulated genes by fold change for treatment groups compared to control, weighted by combination, and **(B)** a cell profiling analysis normalized via an abundance score. DC, dendritic cells; NK, natural killer cells; Th1, T helper 1 cells; Tregs, regulatory T cells.

To understand the mechanism underlying the increase in tumor control following docetaxel and mSTAR1302 therapy, gene expression data were collected from murine tumors in each treatment cohort. Among the top differentially downregulated genes, CDK1 (a cell cycle regulator) was associated with cell cycle arrest and tumor suppression (24) (**Figure 4A**). Other downregulated genes included DLL4, a regulator of NOTCH signaling, and CCL28, a chemokine important for mucosal immunity, both of which have been shown to promote angiogenesis and tumorigenicity (25–27) (**Figure 4A**). Pro-inflammatory genes such as CCL17 and CFD were among the top differentially upregulated genes (28, 29) (**Figure 4A**). CD160 and Klrc2, essential for NK cell activation and response, were also upregulated in mice receiving mSTAR1302 monotherapy or combination treatment (30, 31) (**Figure 4A**). Lastly, cell type profiling was used to characterize the different immune cell populations within the tumor, represented by an abundance score or the relative abundance of each cell type, estimated by the gene expression profile of cell type-specific gene markers. Notably, cytotoxic cells of all types were most upregulated in tumors treated with the combination of docetaxel and mSTAR1302 (**Figure 4B**). Other cell types that were upregulated with mSTAR1302 or combination treatments included the murine equivalent of CD56^dim^ NK cells (characterized as CD27- CD11b+), Th1 cells, dendritic cells, macrophages, B cells, CD4 T cells, and CD8 T-cells (**Figure 4B**). mSTAR1302 treatment also increased regulatory T cell (Treg) recruitment in both monotherapy and combination-treated tumors. Similarly, both the mSTAR1302 and combination-treated cohorts showed increased CD8 exhaustion (**Figure 4B**). Together, these data indicated a decrease in genes associated with cell cycle progression, angiogenesis, and pro-tumorigenic signaling pathways, as well as an increase in inflammatory processes and infiltration by cytotoxic immune cells in tumors of mice treated with mSTAR1302 or the combination of docetaxel and mSTAR1302.

### 3.6 Vβ13^+^ TILs expand with docetaxel and mSTAR1302 combination treatment in the 4T1 triple-negative breast cancer model

**Figure 5:**
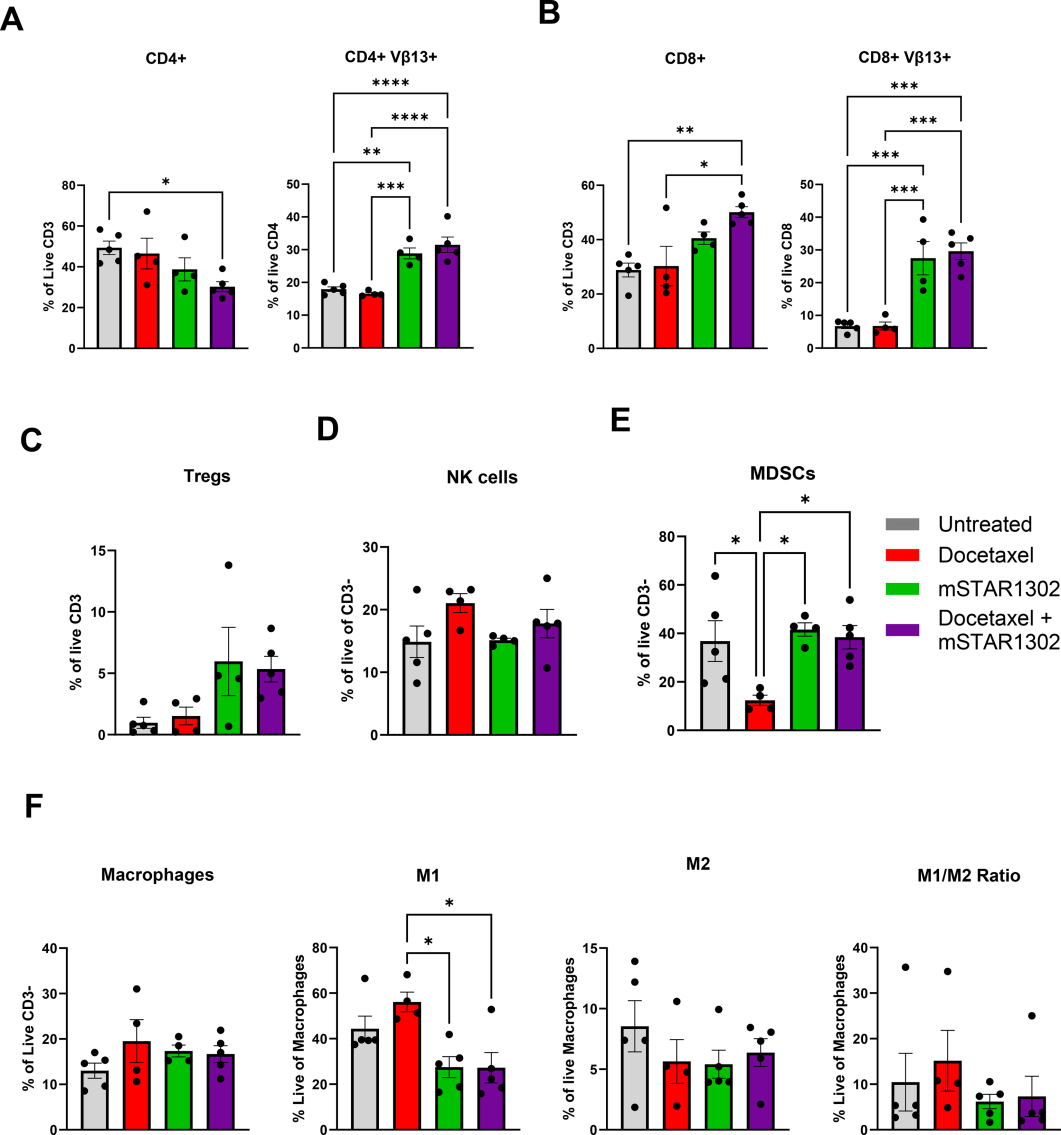
Vβ13 T cells are upregulated with docetaxel and mSTAR1302 combination treatment in 4T1 triple negative breast cancer model. Balb/c mice were inoculated with 4T1 cells on day 0 and treated with mSTAR1302 and docetaxel as previously described in **Figure 1A**. Tumors were harvested, processed, and stained on day 23. Flow cytometry was performed to determine the frequency of **(A)** CD4 and **(B)** CD8 T cells, which were further analyzed for expression of Vβ13 (Vβ8.1), **(C)** Tregs, **(D)** NK cells, **(E)** MDSCs, and **(F)** macrophages, which were further analyzed to determine M1, M2, and M1:M2 status. Statistical tests: comparison between groups: one-way ANOVA with Tukey’s *post hoc* test. Error bars, SEM. *p*<*0.05, **p*<*0.01, ***p*<*0.001, ****p*<*0.0001. ANOVA, analysis of variance; MDSCs, myeloid-derived suppressor cells; NK, natural killer cells; Tregs, regulatory T cells.

STAR0602 and its murine surrogate mSTAR1302 have been studied for their monotherapy effects on immune signaling. However, the immune landscape of tumors post-mSTAR1302 treatment in combination with docetaxel has yet to be elucidated. Tumor infiltrates from 4T1 tumors on day 23 were analyzed via flow cytometry. It was observed that tumors receiving mSTAR1302 treatment had a significant increase in CD4^+^ Vβ13^+^ T cells compared to untreated tumors (p*<*0.01) and docetaxel-treated tumors (p*<*0.001). Similarly, CD4^+^ Vβ13^+^ T cells were significantly upregulated in mice treated with the combination therapy compared with untreated (p*<*0.0001) and docetaxel-treated (p*<*0.0001) mice (**Figure 5A**). Overall, CD8^+^ T cells were upregulated in the cohort receiving the combination therapy compared to the untreated group (p*<*0.01) and the docetaxel group (p*<*0.05). In addition, CD8^+^ Vβ13^+^ T cells were significantly increased within the docetaxel and mSTAR1302 combination group compared to untreated (p*<*0.001) and docetaxel-treated (p*<*0.001) cohorts, which was also observed with mSTAR1302 monotherapy (**Figure 5B**). There were no changes in the frequency of Tregs or NK cells between treatment groups (**Figures 5C, D**). 4T1 tumors are well characterized for their abundance of myeloid-derived suppressor cells (MDSCs) within the tumor and tumor microenvironment (32, 33). The combination of docetaxel and mSTAR1302 did not affect the MDSC population (**Figure 5E**). There were also no notable changes in macrophage frequency, M1 polarization, M2 polarization, or the M1:M2 ratio in the combination-treated cohort compared to the untreated group (**Figure 5F**).

### 3.7 Docetaxel and mSTAR1302 antitumor immune responses are dependent on CD4, CD8, and NK cell activity

**Figure 6:**
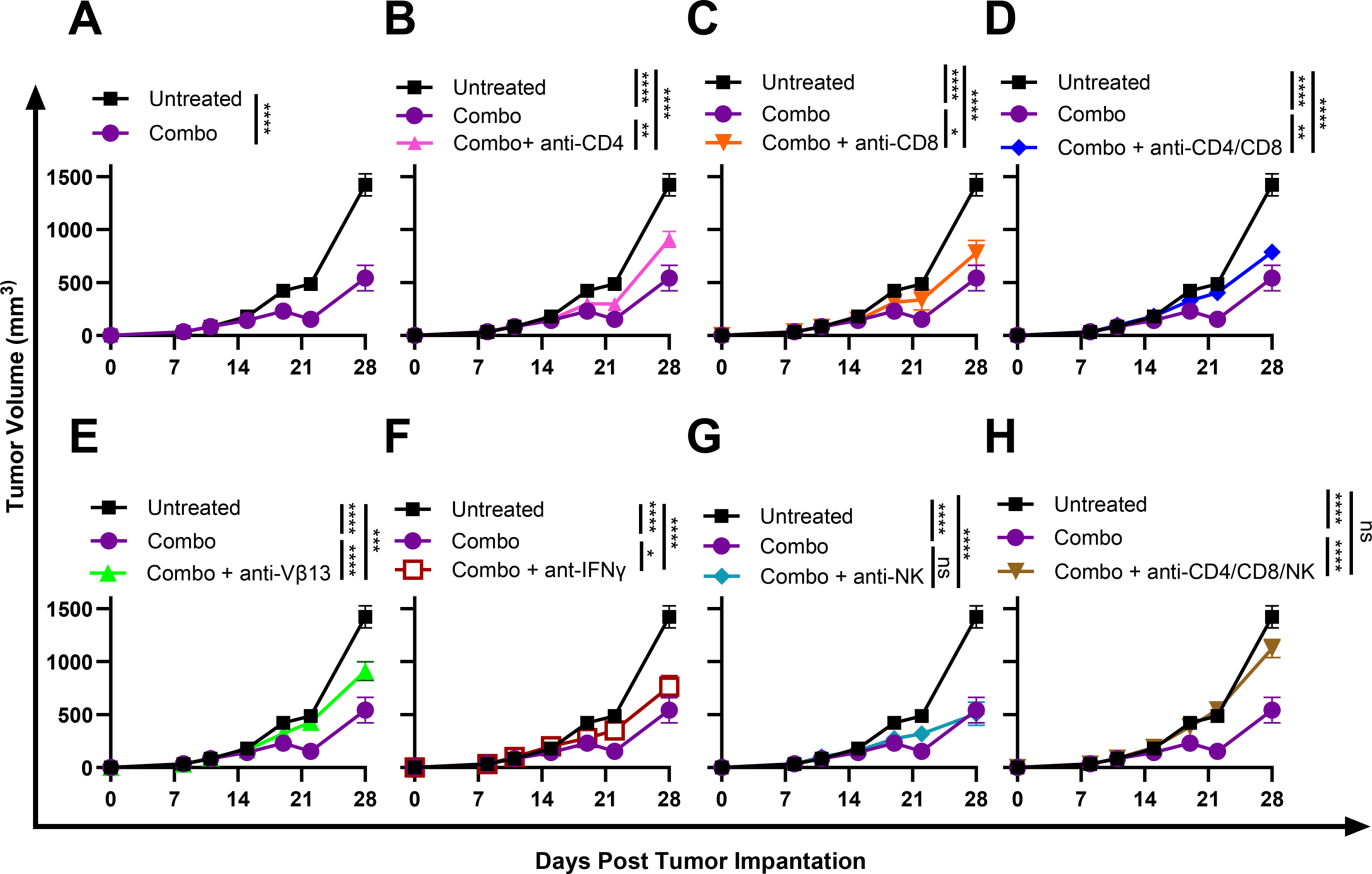
Docetaxel and mSTAR1302 antitumor immune response dependent on CD4+ T, CD8+ T, and NK cell activity. Female Balb/c mice were implanted with 4T1 tumors and treated as described in **Figure 1A** with docetaxel and mSTAR1302. **(B, D, H)** anti-CD4 (100 μg, i.p.) and **(C, D, H)** anti-CD8 depleting antibodies were administered on days 6, 7, and 8, post-tumor implantation and then once every 7 days thereafter. **(E)** an anti-Vβ13 (1 mg/kg, i.p.) depleting antibody was administered on days 6 and 8, and every 7 days thereafter. **(F)** anti-IFN-γ (100 μg, i.p.) was administered 2 days prior to, the same day as, and 2 days following mSTAR1302 treatment (i.e., days 7, 9, 11, 14, 16, 18, 21, 23, and 25). **(G, H)** anti-NK–depleting antibody (25 µl in 100 µl of PBS, i.p.) was administered on days 5 and 7 and then every 5 days subsequently. **(A–H)** mean tumor volume displayed. Statistical tests: tumor growth: two-way ANOVA with Tukey’s *post hoc* test; error bars, SEM. *p*<*0.05, **p*<*0.01, ***p*<*0.001, ****p*<*0.0001. ANOVA, analysis of variance; anti, antibody; IFN-γ, interferon gamma; i.p., intraperitoneal; ns, not significant; NK, natural killer.

The contribution of key immune cell populations to the antitumor effects of docetaxel and mSTAR1302 combination treatment was assessed via the depletion of varying immune cell types or molecules. The control group of mice received no treatment, while all other experimental groups received a regimen of both docetaxel and mSTAR1302 plus varying depleting antibodies. Mice treated with the combination of docetaxel and mSTAR1032 showed a significant increase in antitumor activity compared to untreated mice (p*<*0.0001) (**Figure 6A**). When CD4^+^ T cells were depleted, an antitumor effect was still observed compared to the control (p*<*0.0001); however, the efficacy was significantly reduced compared to combination-treated mice (p*<*0.01) (**Figure 6B**). Similar results were observed with CD8^+^ T-cell depletion (**Figure 6C**) and with co-depletion of CD4+ and CD8+ T cells (**Figure 6D**). Depletion of Vβ13 also caused a partial reduction in antitumor activity compared to the combination-treated group (p*<*0.0001) (**Figure 6E**). IFN-γ depletion resulted in a decrease in antitumor efficacy compared to the combination of docetaxel and mSTAR1302 (p*<*0.05) (**Figure 6F**). NK cell depletion did not affect tumor volume compared to the combination (p*<*0.05) (**Figure 6G**). However, when CD4+ T, CD8+ T, and NK cells were depleted concurrently, all antitumor activity was abrogated compared to the combination (p*<*0.0001), resulting in no significant difference between the triple-depleted group and the untreated group (**Figure 6H**). These data, taken together, indicate that CD4+ T, CD8+ T, and NK cells are all necessary for the immune-driven antitumor activity observed with the combination treatment of docetaxel and mSTAR1302. These findings indicate that the antitumor activity of combination therapy is not driven by a single immune subset but instead reflects coordinated interactions between CD4+ T, CD8+ T, and NK cells.

### 3.8 Docetaxel and mSTAR1302 co-treatment promotes antigen-specific T cells in the 4T1 tumor model

**Figure 7:**
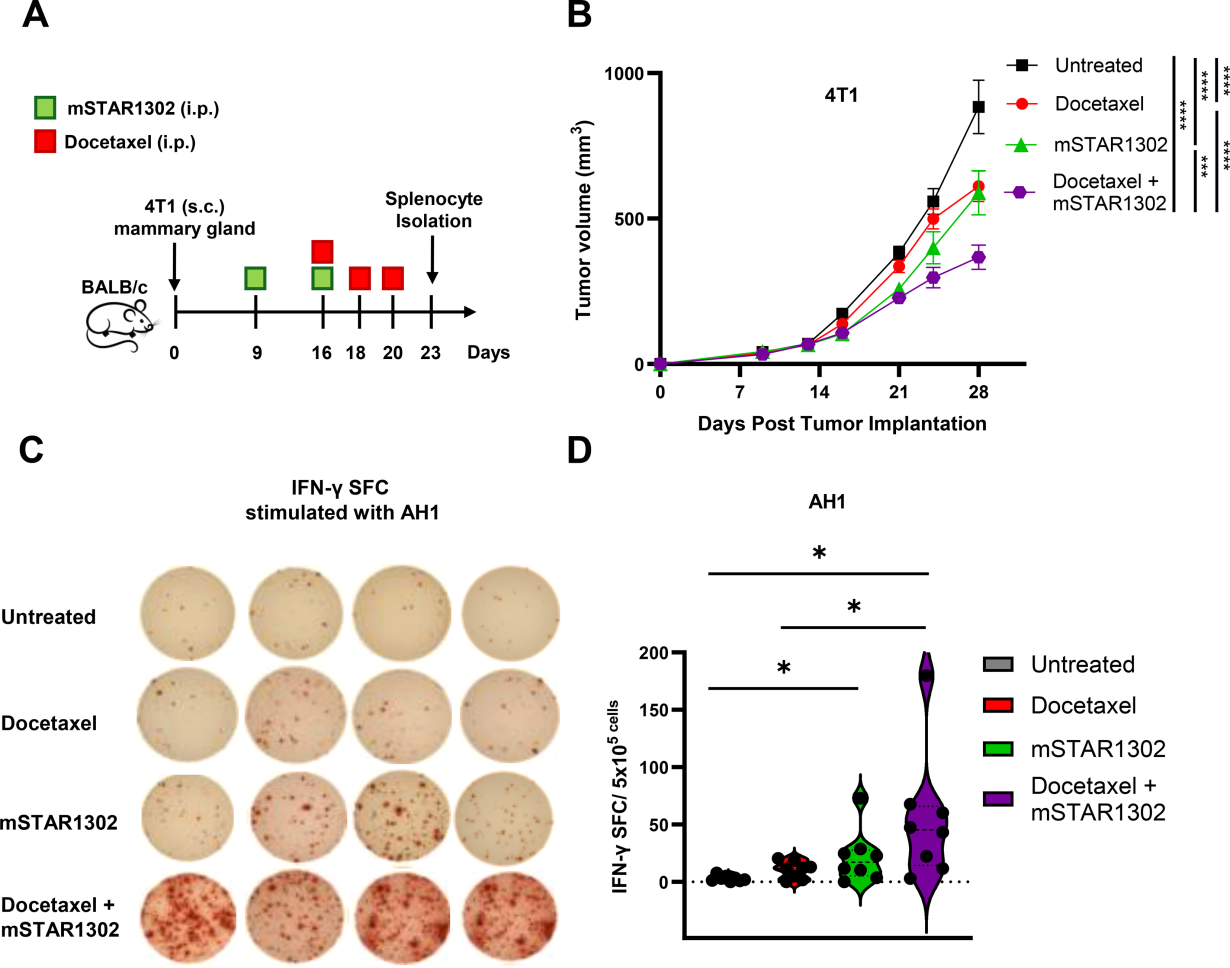
Docetaxel and mSTAR1302 co-treatment promote antigen-specific T cells in the 4T1 tumor model. Female Balb/c 4T1 tumor-bearing mice were treated with mSTAR1302 and docetaxel as previously described (n=20/group). **(A)** pictogram depicts experimental design. **(B)** mean and individual tumor volumes recorded over time. On day 23, the spleens of 8 mice per group were surgically excised and processed. Splenocytes were plated on ELISPOT plates coated with IFN-γ–specific capture antibody and co-cultured with AH1 peptide, CD3/CD28 antibody as a positive control, or β-gal overnight, then developed with detection antibody. β-gal was used to normalize data to account for non-specific binding and background. ELISPOT plates were pictured **(C)** and quantified **(D)** for total number of spots. Statistical tests: tumor growth: two-way ANOVA with Tukey’s *post hoc* test. Comparison between groups: one-way ANOVA with Tukey’s *post hoc* test. Error bars, SEM. *p*<*0.05, ***p*<*0.001, ****p*<*0.0001. ANOVA, analysis of variance; IFN-γ, interferon gamma; i.p., intraperitoneal; s.c., subcutaneously; SFC, spot-forming cells.

To assess the impact of docetaxel and mSTAR1302 co-treatment on the induction of antigen-specific T cells, female Balb/c mice were injected with 4T1 tumor cells in the mammary fat pad and treated as depicted in **Figure 7A**. Again, it was demonstrated that mice treated with a combination of docetaxel and mSTAR1302 exhibited the greatest tumor control compared with all other treatment groups (**Figure 7B****).** On day 23, eight mice were removed from the study and their spleens harvested. An ELISPOT assay was performed to assess splenocyte activation (via IFN-γ production) following stimulation with AH1, a well-characterized endogenous tumor-associated antigen expressed in 4T1 cells that binds to MHCI (34). Splenocytes from mSTAR1302-treated mice had an increase in spot-forming cells (SPC) compared to untreated mice (p*<*0.05) after overnight stimulation with AH1 (**Figures 7C, D**). Combination-treated mice had the highest upregulation of SPC, which was significantly higher compared to untreated (p*<*0.05) and docetaxel-treated mice (p*<*0.05) (**Figures 7C, D**).

## 4 Discussion

In contrast to traditional anti-CD3ϵ TCR agonists employed in bispecific T-cell engager (TCE) molecules, STAR0602 is a novel, first-in-class bifunctional T cell agonist that combines a selectively acting TCR agonist with a T-cell costimulatory moiety that acts in *cis* on the same T cell to promote the selective activation and expansion of Vβ6/Vβ10 TCR-expressing T cells that adopt a memory-like effector phenotype. mSTAR1302, the murine surrogate for STAR0602, has been shown to exert monotherapy activity in several tumor models (21). Unlike conventional CD3-directed T-cell engagers, STAR0602 selectively targets defined Vβ subsets and delivers IL-2 costimulation in *cis*, enabling expansion of functionally relevant T-cell populations rather than broad, non-specific activation. This distinction may contribute to the coordinated immune responses observed in this study.

Here, we demonstrate that docetaxel enhances the antitumor activity of a Vβ-targeted bifunctional T-cell agonist through a mechanism involving tumor sensitization and coordinated immune activation. Specifically, our data identify TRAIL-R2–dependent signaling as a critical axis through which chemotherapy-induced immunogenic modulation enhances immune-mediated tumor clearance.

We show that the combination of docetaxel and mSTAR1302 increased antitumor activity and survival compared to single agents in breast and prostate tumor models (**Figures 1**, **6**), supported by an increase in cytotoxic T cells and NK cells (**Figure 5**) and the upregulation of pro-apoptotic signaling molecules (**Figure 4**). We also demonstrated that the combination reduced lung metastases in the 4T1 tumor model (**Figure 1**), which is associated with the downregulation of genes crucial for angiogenesis and tumorigenesis (**Figure 4**). It is important to note that when comparing the mSTAR1302 monotherapy to docetaxel and the mSTAR1302 combination, most flow cytometry and gene expression data between the two groups followed similar trends in the upregulation and downregulation of genes, pathways, and immune-cell populations, confirming that most changes within the immune system are driven by mSTAR1302 rather than docetaxel. This was similarly observed in Rosato et al., following the combination of mSTAR1302 with cisplatin and checkpoint blockade, where mSTAR1302 and the triplet therapy showed similar trends in immune cell expression within the tumor and tumor microenvironment (22). These data suggest complementary mechanisms in which mSTAR1302 drives T-cell expansion, while docetaxel enhances tumor susceptibility through FAS and TRAIL signaling.

T-cell engagers have traditionally been used to treat hematological malignancies and have more recently been explored for the treatment of solid cancers. In 2022, tebentafusp, the first bispecific TCE approved by the U.S. Food and Drug Administration (FDA) for the treatment of solid tumors, was approved for the treatment of melanoma (35). Shortly thereafter, tarlatamab was approved for the treatment of relapse/refractory extensive-stage small cell lung cancers following chemotherapy treatment (36), while several others are still under investigation in clinical trials (37). Many preclinical studies are exploring TCEs in combination strategies. A survey by Sano et al. investigated the use of a bispecific redirecting antibody, ERY974, targeting CD3 and glypican-3, in combination with multiple chemotherapies in cold, or “non-inflamed,” tumors. The authors concluded that ERY974, in combination with the chemotherapeutic agents paclitaxel or cisplatin, exhibited significant antitumor activity in the NCI-H446 non-small-cell lung cancer murine model compared with the single agents. Similar results were observed in mice inoculated with MKN-45, a human gastric adenocarcinoma cell line, when treated with ERY974 and capecitabine (38). In another study conducted by Wathikthinnakon et al., the chemotherapeutic gemcitabine was evaluated in combination with a bispecific TCE targeting PD-L1 and CD3 to treat cholangiocarcinoma, an aggressive cancer of the bile duct (39). The data showed that gemcitabine, in combination with TCEs, increased T-cell cytotoxicity against cholangiocarcinoma cell lines. Most recently, Rosato et al. explored the use of mSTAR1302 in HNSCC with cisplatin and anti-PD1. Their studies showed that triple therapy was more efficacious than any single agent or any combination of two agents. Furthermore, it was shown that mSTAR1302 resensitized tumors to standard-of-care cisplatin and anti-PD1 therapy after first-line therapy failure. This study, while similar to the data presented here, differs significantly in the antitumor mechanisms. In Rosato et al., the main driver of antitumor activity was an increase in cytotoxic TILs in the tumor microenvironment, but, more importantly, the upregulation of pro-inflammatory cytokines, with an emphasis on the role of IFNγ in antitumor activity (22).

Our study expands on the observations of Sano et al. (38), Wathikthinnakon, et al. (39), and Rosato et al. (22), and extends the benefits of combining TCEs with chemotherapeutics in tumors with low immune-cell infiltration. Furthermore, our data show that this combination can be effective even without direct targeting of antigen-specific T cells, thereby elucidating the potential therapeutic use of STAR0602 and docetaxel across many tumor types, irrespective of tumor-associated antigens.

Although docetaxel is commonly recognized for its cytotoxic effects as a chemotherapeutic agent, its ability to modulate the immune response and synergize with immunotherapies is well established (12, 16–18). These phenotypic changes include upregulation of antigen presentation, MHCI, for tumor-associated antigens, costimulatory molecules, activating NK cell ligands, and death receptors (23). We previously reported docetaxel’s ability to cause the *in vitro* expression of immunogenic molecules such as TRAIL-R2 and FAS, which we confirmed and recapitulated both *in vitro* with tumor cells treated with docetaxel (**Figure 2A**) and *in vivo* in mice treated with docetaxel and/or mSTAR1302 (**Figures 2C, D**). However, docetaxel monotherapy was insufficient at inducing upregulation of TRAIL-R2 in 4T1 tumors (**Figure 2D**). This lack of *in vivo* expression of TRAIL-R2 following docetaxel treatment may be attributed to 4T1 tumors being immune-excluded and highlights the ability of mSTAR1302 and docetaxel to synergistically enhance lymphocyte infiltration within the tumor and upregulate death receptor signaling. Furthermore, docetaxel treatment of tumor cell lines functionally enhanced TRAIL receptor, FAS, and NK-mediated tumor lysis (**Figures 2B, F**). Additionally, antitumor activity was reduced in the 4T1 model and completely abrogated in the TRAMP-C2 model when TRAIL-R2 expression was knocked down. While TRAIL-R2 knockdown attenuated or abrogated therapeutic efficacy, the extent of this effect differed between tumor models. In TRAMP-C2 tumors, near-complete knockdown of TRAIL-R2 resulted in complete loss of response, whereas in 4T1 tumors, partial knockdown was associated with only partial attenuation of efficacy. Although this may reflect differences in knockdown efficiency, it is also likely influenced by tumor-intrinsic factors, including potential redundancy in death receptor pathways such as FAS signaling, as well as differences in tumor microenvironment. Additionally, these studies were conducted using a single CRISPR-derived clone per cell line, and clonal variability cannot be excluded. Future studies incorporating multiple independent clones or orthogonal approaches to modulate TRAIL-R2 expression will be important to further validate these findings.

TRAIL-R2 and FAS are death receptors and members of the tumor necrosis factor (TNF) receptor superfamily, which, in addition to cell death, have multifaceted roles within the immune system (40). Activation of these receptors by their corresponding ligands, TRAIL and FASL, induces intrinsic apoptotic signaling. Although activated by different ligands, TRAIL-R2 and FAS signaling pathways converge at the binding of the death-inducing signaling complex (DISC), which recruits the adaptor protein, FAS-associated death domain (FADD), and subsequently recruits caspase-8/caspase-10, initiating the caspase cleavage cascade. Death receptor signaling has been exploited for its potential to treat cancer through multiple mechanisms, including molecules that upregulate receptor expression, receptor agonists, and drugs that resensitize tumor cells to TRAIL- or FAS-mediated apoptosis (41, 42). Our study explored the role of docetaxel pretreatment on the expression of FAS and TRAIL-R2 (**Figure 2A**). In addition to our data on docetaxel, several other commonly used chemotherapeutic agents, including the taxane paclitaxel, have been shown to upregulate TRAIL-R2 expression across a broad array of prostate cancer cell lines and to improve antitumor activity when used in combination with TRAIL (41, 42). Many recent studies have focused on using TRAIL-R2– or FAS-inducing drugs in combinations with other therapeutics. A recent report discussed the use of a specific epitope of FAS, which effectively targets ovarian tumor cells and could improve the standard effect when used in combination with chimeric antigen receptor T-cell therapy (CAR-T) (43). A different study designed a bifunctional antibody against PD-L1 and TRAIL that increased apoptosis induction across multiple cell lines (44). These studies, among many others, highlight the therapeutic benefits of using drugs that upregulate death receptor signaling pathways as part of combination strategies because of their ability to enhance the effectiveness of several other therapeutic agents and increase the induction of apoptosis in many cancer types. Furthermore, our work highlights the ability of mSTAR1302 monotherapy to upregulate TRAIL-R2 and FAS *in vivo*, which is further increased when used in combination with docetaxel (**Figure 2B**). We observed that treatment with docetaxel- and mSTAR1302-mediated tumor regression in the 4T1 syngeneic tumor model required the activity of CD4^+^ T, CD8^+^ T, and NK cells (**Figure 6**). Depletion of any single population had only minimal effects on antitumor activity, whereas concurrent depletion of CD4^+^ T, CD8^+^ T, and NK cells suppressed all antitumor activity. Conversely, in a previous study using the EMT6 breast cancer model, the antitumor activity of mSTAR1302 monotherapy was abrogated with the depletion of Vβ13, CD8^+^ T cells, or CD4^+^ T cells (21). In 4T1 tumor-bearing mice, flow cytometry data showed no significant changes in NK cell frequency within the tumor following the different treatment regimens (**Figure 5B**). However, the cell profiling analysis revealed an increase in a population of cytotoxic murine NK cells similar to human CD56^dim^ NK cells in tumors treated with docetaxel and mSTAR1302. We hypothesize that, in our model, the antitumor activity attributed to mSTAR1302 is not solely due to an expansion of Vβ13 T cells, but that the expansion of Vβ13 T cells may serve as a driver of the polyclonal expansion of T cells, explaining why depletion of Vβ13+ cells did not fully attenuate the antitumor activity of the combination (**Figure 5B**). Furthermore, these data suggest that bidirectional crosstalk may occur between T and NK cells, promoting their overall activation and function. Although no significant differences in Treg frequency were observed across treatment groups, frequency alone may not fully capture the immunoregulatory role of these cells. Immunogenic modulation has been shown to alter Treg suppressive function independently of changes in abundance. Therefore, it remains possible that functional changes in Tregs contribute to the observed therapeutic effects. Future studies assessing Treg suppressive capacity and phenotypic markers will be important to further define their role in this context. Together, these data support a model in which docetaxel-induced tumor sensitization via TRAIL-R2 enhances susceptibility to cytotoxic signaling, while mSTAR1302-driven expansion of Vβ13+ T cells promotes coordinated CD4+ T, CD8+ T, and NK cell–mediated tumor clearance. An additional observation from the RNA profiling analysis (**Figure 4**) was the increase in markers associated with CD8 T-cell exhaustion following mSTAR1302 and combination treatment. While this may reflect a consequence of robust T-cell activation and expansion, sustained antigen exposure and continuous stimulation could drive the emergence of an exhausted phenotype over time. This state is typically characterized by reduced effector function and upregulation of inhibitory receptors, which may ultimately limit the durability of antitumor responses despite strong initial tumor control. These findings suggest that, although mSTAR1302 promotes effective T-cell activation, prolonged signaling may also necessitate mechanisms to sustain T-cell functionality. This raises the possibility that combination strategies incorporating checkpoint blockade or other approaches to reinvigorate exhausted T cells could further enhance long-term therapeutic efficacy.

STAR0602 is currently being evaluated as an infusion given every 2 weeks in a Phase 1/2 clinical trial (STARt-001) in patients with solid, PDL1-resistant, antigen-rich tumors. Preliminary Phase 1 data identified a well-tolerated dose level for evaluation in a Phase 2 efficacy part, where monotherapy activity in patients with high mutational burden tumors who received the optimal biological dose (0.8-1.2 mg/kg) was also demonstrated (45). Here, we utilized the 4T1 breast cancer and TRAMP-C2 prostate cancer models, both characterized as immunologically “cold” tumors (46). In addition, 4T1 tumors are resistant to checkpoint inhibitors, such as anti-PD1 and anti-cytotoxic T-lymphocyte-associated protein-4 (CTLA-4) (47). TRAMP-C2 tumors are sensitive to checkpoint inhibition but show increased sensitivity when combined with other treatment modalities (48). Both the 4T1 and TRAMP-C2 models demonstrated monotherapy activity with mSTAR1302, which was enhanced by the addition of docetaxel. Although a subset of mice achieved complete tumor regression during treatment, tumor recurrence was observed following cessation of therapy, indicating that durable tumor control and long-term immunological memory were not fully established. The absence of tumor re-challenge or long-term follow-up studies represents a limitation of the current work. Future studies evaluating tumor re-challenge and memory T-cell responses will be important to determine whether this therapeutic approach can induce durable protective immunity, particularly in immunologically “cold” tumor models. It is also informative to consider how this approach compares with adoptive cellular therapies such as CAR T-cell therapy. CAR T-cell therapies rely on the persistence and expansion of engineered T cells to mediate durable tumor control and have shown substantial success in hematologic malignancies but remain challenging in solid tumors due to antigen heterogeneity, impaired trafficking, and an immunosuppressive tumor microenvironment. In contrast, the combination of docetaxel and mSTAR1302 is designed to promote coordinated endogenous immune activation together with tumor sensitization through upregulation of death receptor signaling. These approaches may ultimately prove complementary rather than mutually exclusive. For example, therapies that enhance tumor susceptibility and immune-cell infiltration, such as docetaxel and mSTAR1302, could potentially improve the efficacy of adoptive cellular therapies in solid tumors.

Furthermore, previous studies have shown monotherapy activity of mSTAR1302 in other breast cancer (EMT6) and prostate (RM1) tumor mouse models (21). Phase 2 clinical evaluation is currently in progress, with plans to expand dosage studies and explore STAR0602 in combination with other therapeutic agents. While the current study was conducted entirely in murine models, the findings are directly relevant to the clinical development of STAR0602 (invikafusp alfa), the human counterpart of mSTAR1302 currently under evaluation in patients with advanced solid tumors. Importantly, preliminary clinical studies have demonstrated evidence of activity in anti-PDL1–resistant tumors, supporting the translational relevance of Vβ-targeted T-cell activation. Future studies incorporating human *ex vivo* systems or patient-derived samples will be valuable to further validate the mechanisms identified here.

These findings support further clinical evaluation of STAR0602 in combination with chemotherapy and suggest that sequencing strategies that optimize tumor sensitization prior to T-cell expansion may enhance therapeutic efficacy, particularly in immune-excluded tumors.

## Data Availability

The raw data supporting the conclusions of this article will be made available by the authors, without undue reservation.
