# Supplementary material for: Docetaxel enhances Vβ-directed T-cell activation and antitumor immunity mediated by a bifunctional TCR agonist in breast and prostate cancer models
Source: Front Immunol. 2026 Jun 3;17:1850760. doi: 10.3389/fimmu.2026.1850760 (PMC13272395; doi:10.3389/fimmu.2026.1850760)
Supplement: Supplementary file 1 [file DataSheet1.pdf]

Supplemental Figure S1

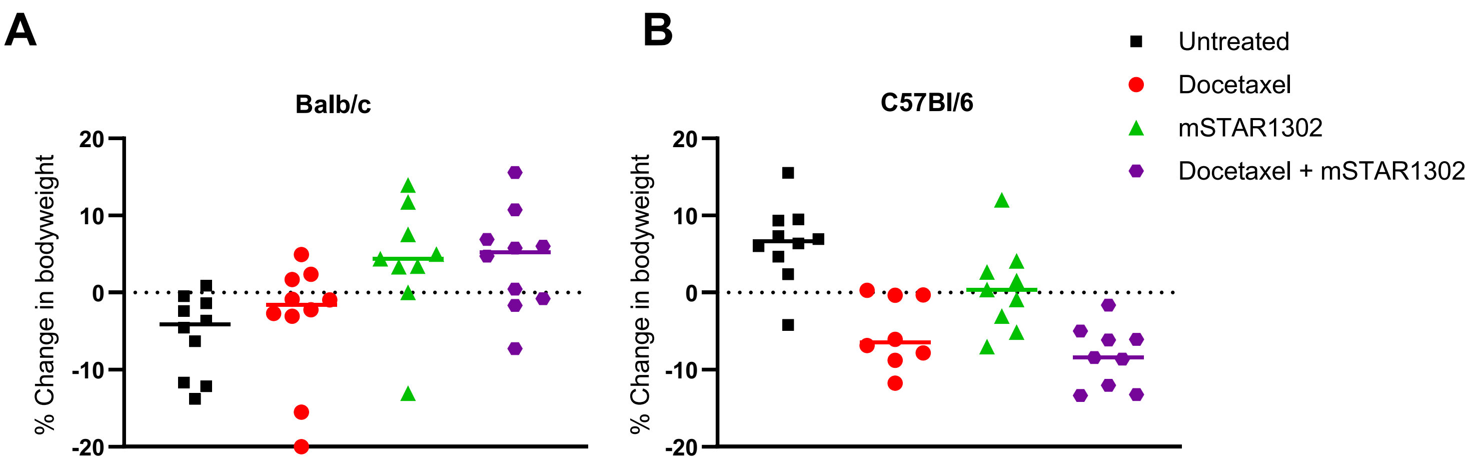

Supplemental Figure S1. Docetaxel and mSTAR1302 are well tolerated in 4T1 and TRAMP-C2 tumor models. **(A)** 4T1 tumor cells were implanted into the mammary fat pad of female Balb/c mice and **(B)** TRAMP-C2 tumor cells were implanted into the right flank of male C57Bl/6 mice. Bodyweight was measured overtime to assess for toxicity and tolerability.

Supplemental Figure S2

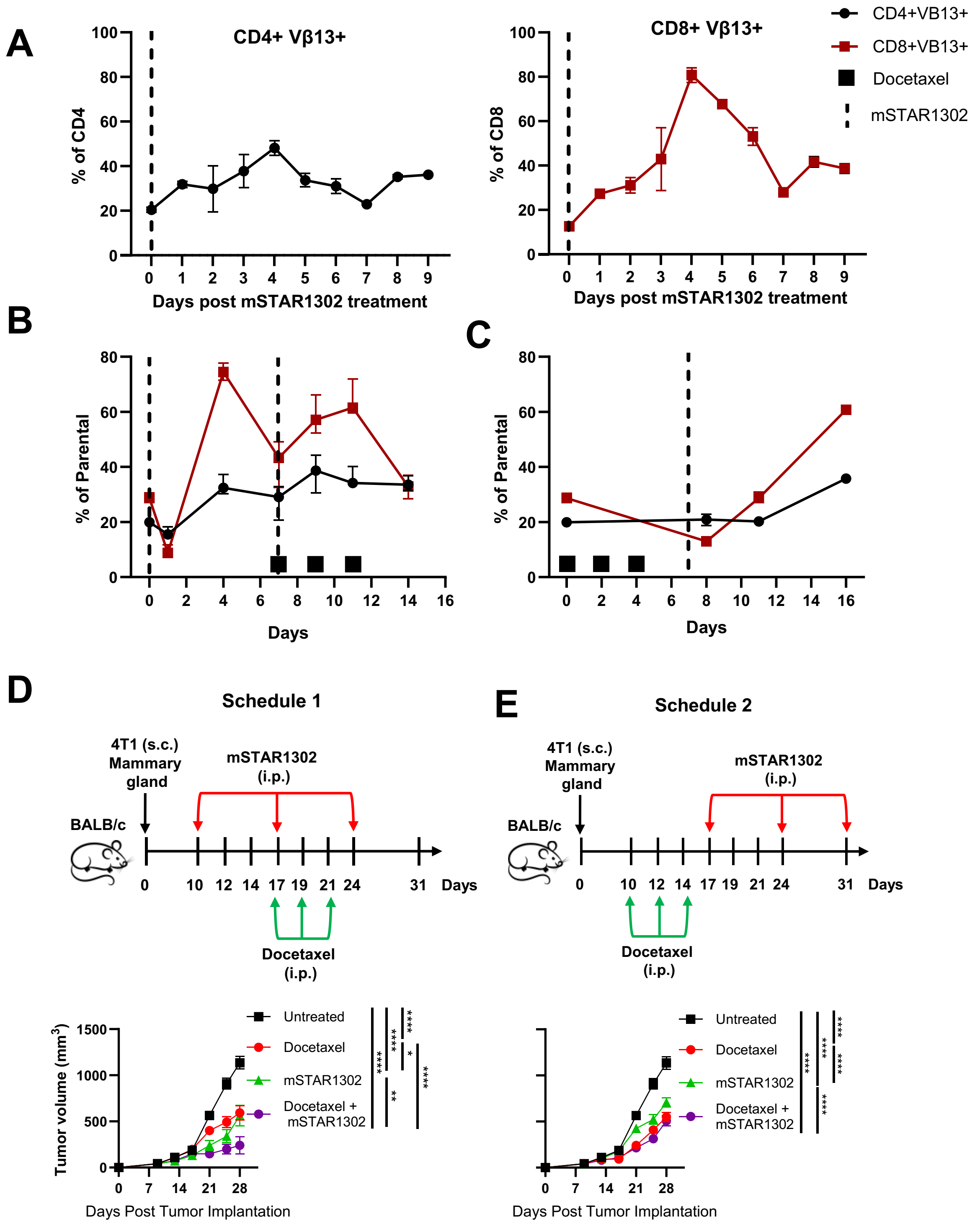

Supplementary Figure S2. Effects of alternate docetaxel and STAR1302 dosing schedules. (A) Non-tumor-bearing female Balb/c mice were treated with a single dose of mSTAR1302 (1mg/kg, i.p.). Spleens from these animals were harvested 0-9 days post-treatment (3 to 4 mice per timepoint) and analyzed by flow cytometry for CD45, CD3, CD4, CD8, and Vβ13 surface markers. **(B,C)** Non-tumor–bearing female Balb/c mice were treated with either (B) mSTAR1302 on days 0 and 7 and docetaxel on days 7, 9, and 11 or (C) docetaxel on days 0, 2, and 4, and mSTAR1302 on day 7. Spleens were harvested between days 0 – 16 post-injection (3 to 4 mice per timepoint) and analyzed via flow cytometry as described above. **(D,E)** Female Balb/c mice were inoculated with 4T1 cells in the mammary fat pad on day 0 and treated with one of two treatment schedules. In Schedule 1 (D), mSTAR1302 (1 mg/kg, i.p.) was administered on days 10, 17, and 24, with docetaxel (250 µg, i.p.) subsequently administered on days 17, 19, and 21. In Schedule 2 (E), docetaxel (250 µg, i.p.) was administered first on days 10, 12, and 14, followed by mSTAR1302 (1 mg/kg) on days 17, 24, and 31. Mean tumor volumes for both schedules were measured. Statistical tests: Tumor growth: two-way ANOVA with Tukey's post hoc test. Error bars, SEM. * $P < 0.05$, ** $P < 0.01$, *** $P < 0.001$, *** $P < 0.0001$.
s.c., subcutaneously; i.p. intraperitoneal; ANOVA, analysis of variance.

Supplemental Figure S3

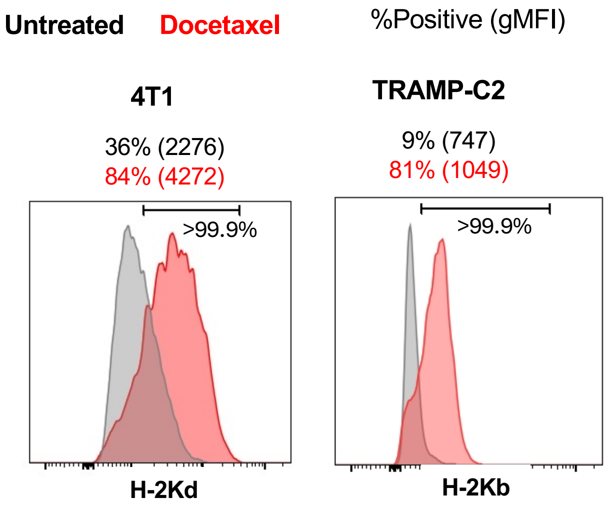

Supplemental Figure S3. MHCI upregulated following docetaxel pre-treatment. 4T1 and TRAMP-C2 tumor cells were pretreated for 72 hours with 250 ng/mL of docetaxel and then stained for flow cytometry. For MHCI markers. MHCI; major histocompatibility complex class I. Comparison between two histograms: Non-parametric Kolmogorov-Smirnov (KS) test.
